# Clinicopathological features and surgical treatment of cervical oesophageal cancer

**DOI:** 10.1038/s41598-017-03593-0

**Published:** 2017-06-12

**Authors:** Shao-bin Chen, Xi-hong Yang, Hong-rui Weng, Di-tian Liu, Hua Li, Yu-ping Chen

**Affiliations:** 1 Department of Thoracic Surgery, Cancer Hospital of Shantou University Medical College, Shantou, Guangdong China; 2 Department of Head and Neck Surgery, Cancer Hospital of Shantou University Medical College, Shantou, Guangdong China

## Abstract

Cervical oesophageal cancer (CEC) is a relatively uncommon malignancy. The biological behaviour and treatment have not been well studied. This retrospective study reviewed the clinicopathological features of 28 patients with CEC who underwent surgical resection to investigate the biological behaviour, treatment and prognosis of CEC. The long-term outcomes of these patients were compared with those of the CEC patients who received definitive chemoradiotherapy and those of thoracic or abdominal oesophageal cancer patients who underwent surgery. The study group contained 21 men and 7 women, ranging in age from 41 to 67 years (median: 56.5 years). The median survival time and the 1-, 3-, and 5-year overall survival rates were 25.0 months, 83.8%, 48.8%, and 41.9%, respectively. Only salvage surgery was found to affect the overall survival (P = 0.007). The long-term outcomes for CEC patients who underwent surgery were significantly better than those who received definitive chemoradiotherapy (P = 0.045) but were similar to those of thoracic or abdominal oesophageal cancer patients. In summary, CEC is an uncommon and aggressive malignancy. The malignant potential of CEC is similar to that of thoracic or abdominal oesophageal cancer. Surgical resection is an important therapeutic strategy and may be associated with better survival rates than definitive chemoradiotherapy.

## Introduction

Cervical oesophageal cancer (CEC) is a relatively uncommon malignancy, accounting for less than 5% of all oesophageal cancers^1^. Treatment options for CEC include primary resection, chemoradiotherapy (CRT), or a combination of the two^2–14^. Historically, surgery has been regarded as the standard treatment for CEC, but the overall survival is rather disappointing^5^. As the proximal cervical oesophagus is adjacent to the larynx, laryngectomy has often been considered to be necessary to achieve radical resection of CEC, but this procedure is not easily tolerated by patients and has a huge impact on the quality of life^4, 15^. Definitive CRT is now the mainstay treatment for CEC according to the National Comprehensive Cancer Network (NCCN) guidelines. However, locoregional disease persists or recurs in 40–60% of the patients who undergo CRT^16, 17^. Due to the low incidence of CEC, their biological behaviour and response to therapies have not been well studied.

Definitive CRT was the standard treatment for CEC patients in our hospital. However, we also performed surgical resection for patients who were willing to undergo surgery since 2001. In the current study, we retrospectively reviewed the clinical characteristics and surgical results of 28 patients with CEC who underwent surgical resection and compared their survival with that of CEC patients who received definitive CRT and that of thoracic or abdominal oesophageal cancer patients who underwent surgery.

## Results

### Patient characteristics

This study group contained 21 men and 7 women, ranging in age from 41 to 67 years (median: 56.5 years). The median length of the primary lesion was 4.0 cm (2.0~7.0 cm). Five patients had multiple lesions in the oesophagus, including two cases of pT1a tumour and one case of pT1b tumour in the middle third of the thoracic oesophagus; and one case of pT1b tumour and one case of pTis tumour in the lower third of the thoracic oesophagus. An R0 resection (complete tumour resection) was achieved in 26 patients (92.9%), and an R2 resection (macroscopic residual tumour) was performed in 2 patients (7.1%). Fifteen patients underwent transhiatal oesophagectomy without thoracotomy, and the other 13 patients underwent thoracoscopic surgery. A mean of 21.4 (range 2–49) lymph nodes was dissected from each specimen, and 14 patients (50%) were confirmed to have lymph node metastases. The postoperative complication rate was 21.4% (6 patients), including one case of wound infection, two cases of anastomotic leakage and three cases of pulmonary complications. No patients died during treatment in the hospital or within 30 days after surgery.

### Survival and prognosis factors

The mean follow-up time of the 28 CEC patients who underwent surgical resection was 34.6 months (range, 2.0–182.0 months). During follow-up, 15 patients died, and no cases were lost to follow-up. The 1-, 3-, and 5-year overall survival (OS) rates for the entire population were 83.8%, 48.8%, and 41.9%, respectively, with a median survival time (MST) of 25.0 months (95% confidence interval (CI) 0.8–49.2 months).

**Figure 1:**
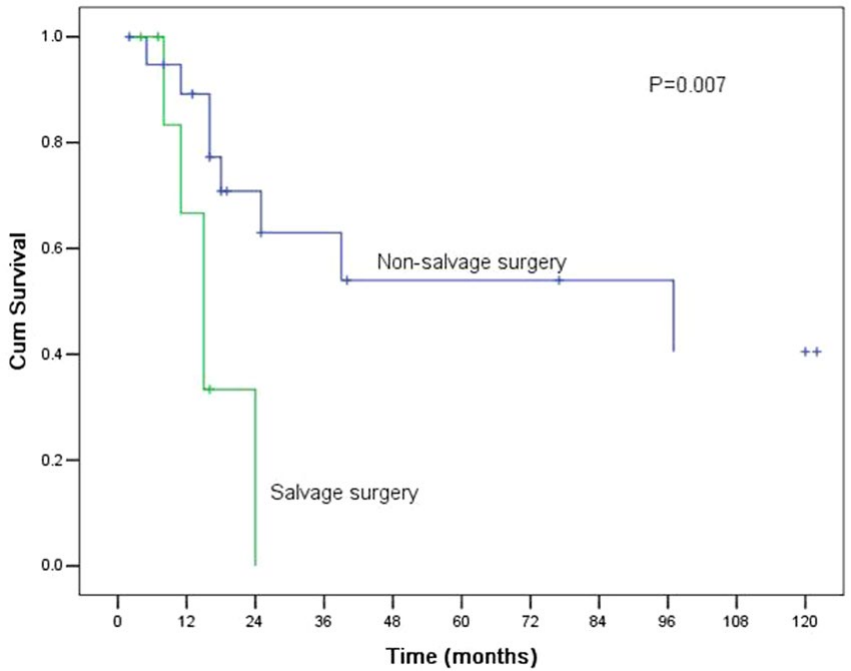
Comparison of the survival curves for patients with cervical oesophageal cancer who underwent salvage surgery and non-salvage surgery. The MST and 5-year OS rate for patients who underwent salvage surgery were 15.0 months (95% CI 10.5–19.5) and 0%, respectively, which were significantly lower than those of 97.0 months (95% CI 0.1–193.9) and 54.0%, respectively, for patients who underwent non-salvage surgery (P = 0.007).

The variables related to survival in univariate analysis are shown in Table 1. Only salvage surgery was found to affect the overall survival (P = 0.007, Fig. 1). The MST and 5-year OS rate for patients who underwent salvage surgery were 15.0 months (95% CI 10.5–19.5) and 0%, respectively, which were significantly lower than those of 97.0 months (95% CI 0.1–193.9) and 54.0%, respectively, for patients who underwent non-salvage surgery. Gender, age, tumour length, tumour cell differentiation, pT category, pN category, pTNM stage, and surgical procedure did not show statistically significant differences in survival (P > 0.05). In multivariate analysis, none of these factors were found to be independent factors (P > 0.05).

**Table 1:** Univariate analysis of the prognosis of 28 patients with cervical oesophageal cancer who underwent surgery.

	Number of patients	MST (months)	5-year OS	*P*-value
Gender				0.744
Male	21	25.0 (1.1–49.0)	40.6%	
Female	7	16.0	50.0%	
Age(years)				0.638
≤60	19	18.0 (3.4–32.6)	41.2%	
>60	9	39.0 (8.2–69.8)	42.9%	
Tumour length				0.102
≤4 cm	15	24.0 (9.0–39.0)	0%	
>4 cm	13	97.0	61.1%	
Tumour cell differentiation				0.107
Well	11	—	77.8%	
Moderate	12	24.0 (13.9–34.1)	27.3%	
Poor	5	15.0 (6.4–23.6)	20.0%	
pT category				0.917
pT_1–2_	7	39.0 (6.2–71.8)	30.0%	
pT_3–4_	21	25.0 (0.0–100.0)	46.3%	
pN category				0.389
pN_0_	14	24.0 (10.0–38.0)	32.3%	
pN_1_	14	97.0 (0.0–206.0)	55.4%	
pTNM stage				0.259
I + II	10	24.0 (14.4–33.6)	16.9%	
III	18	97.0 (0.0–254.7)	57.9%	
Surgical procedure				0.417
Transhiatal	15	18.0 (8.5–27.5)	40.0%	
Thoracoscopy	13	39.0 (14.8–63.2)	30.0%	
Salvage surgery				0.007
Yes	8	15.0 (10.5–19.5)	0%	
No	20	97.0 (0.1–193.9)	54.0%	

*Abbreviations:* MST, median survival time; OS, overall survival; pT category, pathologic T category; pN category, pathologic N category; and pTNM stage, pathologic TNM stage. The TNM stage was based on the sixth edition of the American Joint Committee on Cancer staging system for oesophageal cancer. Surgical procedures were subdivided into two groups: transhiatal oesophagectomy without thoracotomy and thoracoscopic oesophagectomy.

### Comparison of outcomes based on treatment

The definitive CRT group of patients with CEC included 278 men and 82 women, ranging in age from 36 to 79 years (median: 55.0 years). A total dose of 50–70 Gy (median 60 Gy) was delivered at a daily dose of 1.8 to 2 Gy. Concurrent chemotherapy with cisplatin plus 5-fluorouracil or docetaxel was repeated every 3 weeks for 2 to 4 cycles. The median length of the primary lesion was 4.0 cm (1.5~8.0 cm). According to the sixth edition of the American Joint Committee on Cancer (AJCC) staging system for oesophageal cancer, this cohortincluded 15 cases of clinical TNM (cTNM) stage I, 102 cases of cTNM stage II, and 243 cTNM stage III. No significant differences in clinicopathological features were found when compared with those of the surgery group (P > 0.05).

**Figure 2:**
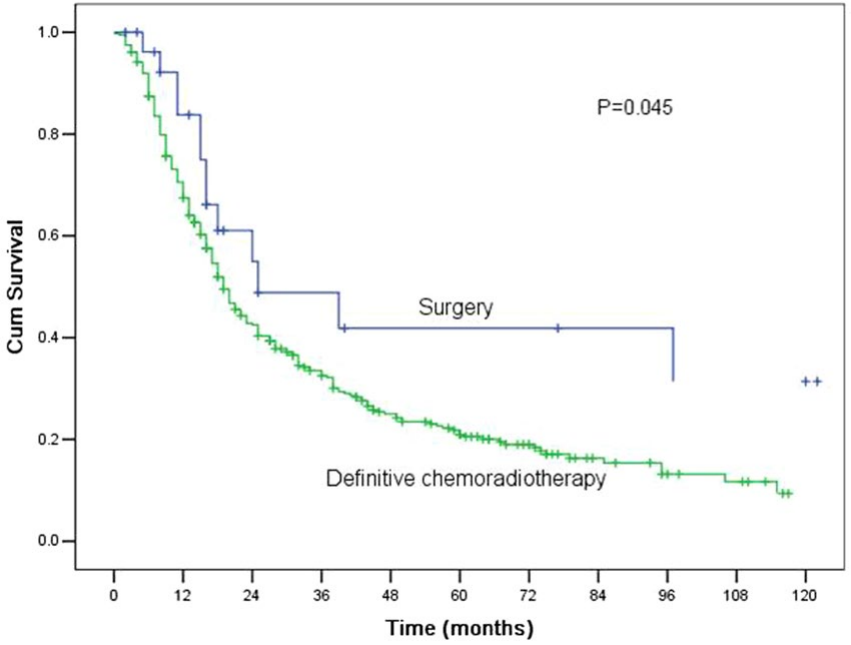
Comparisons of the survival curves for patients with cervical oesophageal cancer who received surgery and definitive chemoradiotherapy. The MST and the1-, 3-, and 5-year OS rates for patients who underwent surgery were 25.0 months (95% confidence interval (CI) 0.8–49.2 months), 83.8%, 48.8%, and 41.9%, respectively, which were significantly better compared with those of 19.0 months (95% CI 16.6–21.4 months), 67.4%, 32.5%, and 21.0%, respectively, for patients who received definitive chemoradiotherapy (P = 0.045).

The MST and the1-, 3-, and 5-year OS rates for the definitive CRT group were 19.0 months (95% CI 16.6–21.4 months), 67.4%, 32.5%, and 21.0%, respectively. The long-term outcomes for CEC patients who received definitive CRT were significantly worse than those who underwent surgery (P = 0.045, Fig. 2).

### Comparison of outcomes based on tumour location

**Figure 3:**
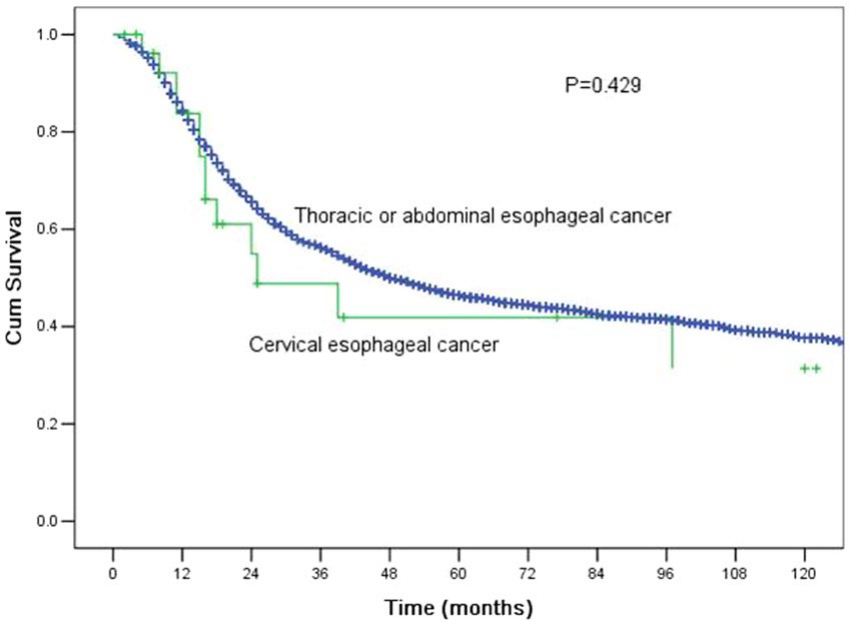
Comparisons of the survival curves for patients with cervical oesophageal cancer and patients with thoracic or abdominal oesophageal cancer who underwent surgery. The median survival time (MST) and the1-, 3-, and 5-year OS rates for CEC patients were 25.0 months (95% confidence interval (CI) 0.8–49.2 months), 83.8%, 48.8%, and 41.9%, respectively, compared with those of 49.0 months (95% CI 43.9–54.1 months), 84.3%, 56.2%, and 46.4% for patients with thoracic or abdominal oesophageal cancer. The survival differences were not statistically significant (P = 0.429).

**Figure 4:**
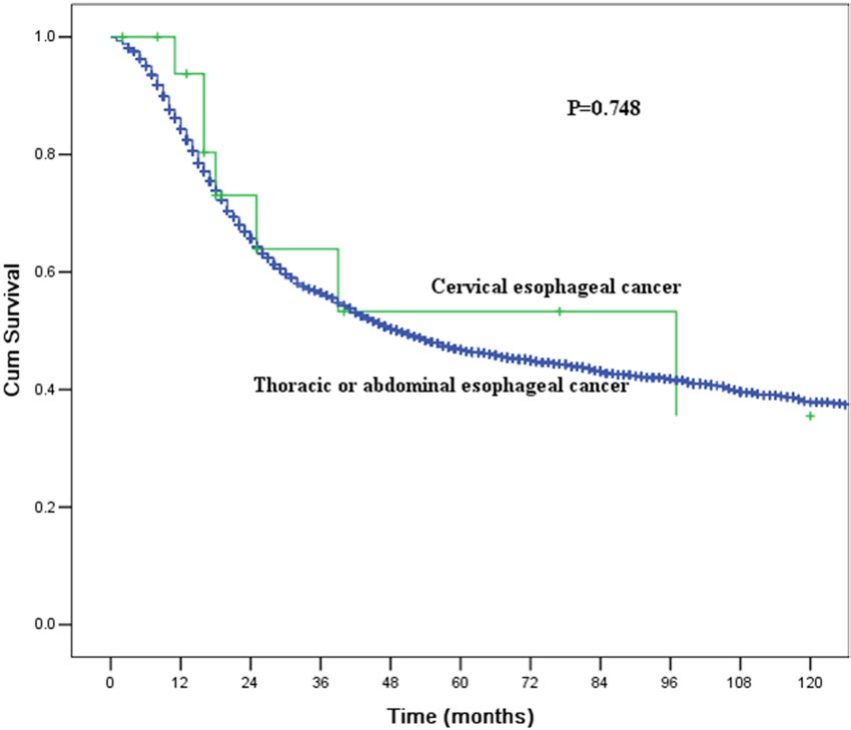
Comparisons of the survival curves for patients with cervical oesophageal cancer and patients with thoracic or abdominal oesophageal cancer who did not receive preoperative therapy. The MST and the1-, 3-, and 5-year OS rates for CEC patients were 97.0 months (95% CI 10.0–184.0 months), 93.8%, 63.9%, and 53.3%, respectively, compared with those of 50.0 months (95% CI 44.1–55.9 months), 84.3%, 56.5%, and 46.8% for patients with thoracic or abdominal oesophageal cancer. The survival differences were not significant (P = 0.748).

The clinicopathological features of the 3,950 patients with thoracic or abdominal oesophageal cancer who underwent surgical resection are shown in Table 2. All factors were similar to those of the patients with CEC, except for preoperative therapy. More patients with CEC underwent preoperative chemoradiotherapy. The MST and the 1-, 3-, and 5-year OS rates were 49.0 months (95% CI 43.9–54.1 months), 84.3%, 56.2%, and 46.4%, respectively. No significant differences were observed in the long-term outcomes between the CEC patients and the thoracic or abdominal oesophageal cancer patients (P = 0.429, Fig. 3). We further compared the long-term outcomes between the CEC patients and the thoracic or abdominal oesophageal cancer patients who did not receive preoperative therapy. The MST and the 1-, 3-, and 5-year OS rates for CEC patients were 97.0 months (95% CI 10.0–184.0 months), 93.8%, 63.9%, and 53.3%, respectively, compared with those of 50.0 months (95% CI 44.1–55.9 months), 84.3%, 56.5%, and 46.8%, respectively, for patients with thoracic or abdominal oesophageal cancer. The survival difference was not significant between these two groups (P = 0.748, Fig. 4).

**Table 2:** Comparison of clinicopathological features between patients with cervical oesophageal cancer and patients with thoracic or abdominal oesophageal cancer who underwent surgery.

	CEC	T/A oesophageal cancer	*P*-value
Gender			1.000
Male	21 (75.0%)	2964 (75.0%)	
Female	7 (25.0%)	986 (25.0%)	
Age(years)			0.697
≤60	19 (67.9%)	2487 (63.0%)	
>60	9 (32.1%)	1463 (37.0%)	
Tumour length			0.079
≤4 cm	15 (53.6%)	1463 (37.0%)	
>4 cm	13 (46.4%)	2487 (63.0%)	
Histology			1.000
SCC	27 (96.4%)	3804 (96.3%)	
Others	1 (3.6%)	146 (3.7%)	
Tumour cell differentiation			0.471
Well	11 (39.3%)	1321 (33.4%)	
Moderate	12 (42.9%)	2129 (53.9%)	
Poor	5 (17.9%)	500 (12.7%)	
pT category			1.000
pT_0–2_	7 (25.0%)	1069 (27.1%)	
pT_3_–_4_	21 (75.0%)	2881 (72.9%)	
pN category			0.850
pN_0_	14 (50.0%)	2092 (53.0%)	
pN_1_	14 (50.0%)	1858 (47.0%)	
pTNM stage			0.187
0–II	10 (35.7%)	1934 (49.0%)	
III–IV	18 (64.3%)	2016 (51.0%)	
Resection margin			0.763
R0	26 (92.9%)	3509 (88.8%)	
R 1–2	2 (7.1%)	441 (11.2%)	
Preoperative therapy			<0.001
None	18 (64.3%)	3559 (90.1%)	
Chemotherapy	0 (0%)	7 (0.2%)	
Radiotherapy	2 (7.1)	193 (4.9%)	
Chemoradiotherapy	8 (28.6)	191 (4.9%)	

*Abbreviations:* CEC, cervical oesophageal cancer; pT category, pathologic T category; pN category, pathologic N category; pTNM stage, pathologic TNM stage; R0, complete tumour resection; R1, microscopic residual tumour; R2, macroscopic residual tumour; SCC, squamous cell carcinoma; and T/A, thoracic or abdominal. The TNM stage was based on the sixth edition of the American Joint Committee on Cancer staging system for oesophageal cancer.

## Discussion

Oesophageal cancer is one of the most aggressive malignancies, and it usually occurs in the thoracic oesophagus. CEC is a very rare disease and is often locally advanced at the time of diagnosis, resulting in limited locoregional disease control and poor survival^14^. Due to the low incidence of this disease, the biological characteristics, treatment, and prognosis were not well studied.

In the current study, we retrospectively analysed the clinicopathological characteristics and outcomes of 28 patients with CEC who underwent pharyngo-laryngo-esophagectomy (PLE) and compared their prognosis with that of the patients with CEC who received definitive CRT and the patients with thoracic or abdominal oesophageal cancer who underwent surgical resection. To our knowledge, few studies have evaluated the outcomes of CEC patients treated with definitive CRT and surgery and even fewer studies have compared the prognosis between patients with CEC and patients with thoracic oesophageal cancer. Only one study by Saeki *et al*.^4^ evaluated the malignant potential between CEC and thoracic oesophageal cancers and found that CEC was not more aggressive. Our study also confirmed that the long-term outcomes were similar between the patients with CEC and patients with thoracic or abdominal oesophageal cancer who underwent surgery. We think that more studies are needed to elucidate the biological characteristics of this cancer.

Historically, surgery has been the standard treatment for CEC. Generally, a PLE is carried out, which includes the resection of the larynx and is considered to have a great risk of major complications as well as a huge impact on quality of life^18^. Nonetheless, novel surgical strategies and improved perioperative management of oesophageal surgery have been developed during the last decades, such as minimally invasive surgery, which might decrease the operative morbidity and mortality. In this study, only 6 patients (21.4%) developed postoperative complications, and no patients died during treatment in the hospital. We think that more careful preoperative evaluation, postoperative critical care, and nutritional support are important in reducing complications in these patients.

Less invasive surgeries such as pharyngo-laryngo-cervical oesophagectomy (PLCE) and larynx-preserving surgery were also performed in patients with CEC in recent years^4, 19^. These types of surgery might achieve functional preservation and improve the quality of life. However, a previous study showed that oesophageal cancer might be a multicentric disease, with a heightened risk of synchronous or metachronous lesions ranging from 12% to 30%^20, 21^. We also found that 17.9% (5/28) of patients had multiple lesions in the thoracic oesophagus in this study, and all of them were pTis or pT1 diseases. The thoracic lesions in two cases were even misdiagnosed in the preoperative examination, as the endoscopy could not be passed across the malignant strictures into the distal oesophagus. This would, therefore, cast serious doubt on the advisability of performing segmental oesophageal resection.

Most of the previous studies have reported disappointing outcomes for patients with CEC due to a delayed diagnosis and the poor performance status of many patients^14^. Definitive CRT is now recommended for CEC to preserve functional upper respiratory and alimentary tracts. However, the reported 5-year overall survival rates ranged only from 18.6% to 37.9%^12, 13, 22–24^. In our study groups, the 5-year OS rate was 21.0% for patients who received definitive CRT, which was similar to that reported in previous studies. Although surgical resection is a more aggressive strategy and has a huge impact on the quality of life, it may provide the best chance of curing oesophageal cancer^25^.

The reported 5-year overall survival rates ranged from 24% to 47.0% for patients with CEC who underwent surgical resection^2, 8, 26–31^. In the current study, the 5-year OS rate was 41.9% for patients who underwent surgical resection. We also found that the long-term outcomes for patients with CEC who underwent surgery were significantly better than those who received definitive CRT. Due to the low number of these studies, more data are needed to confirm our findings. Moreover, neoadjuvant CRT has been increasingly used for the treatment of oesophageal cancer patients, and it has shown a significant survival benefit^32^. We think that further research should be conducted to establish the value of this new therapeutic approach for patients with CEC.

In conclusion, CEC is an uncommon and aggressive malignancy. The malignant potential of CEC is similar to that of thoracic or abdominal oesophageal cancer. Surgical resection is an important therapeutic strategy and may be associated with better survival rates than definitive CRT. As randomized controlled trials for this rare disease are currently impractical, we think that a prospective, multinational database to track these cases will enable a better investigation of the biological behaviour, treatment and prognosis of this disease.

## Materials and Methods

### Patient population

This study was undertaken at the Cancer Hospital of Shantou University Medical College and was approved by the Ethics Committee of that hospital. All methods were carried out in accordance with the approved guideline, and written informed consent from the patients or their family was not deemed necessary for this kind of retrospective study by the Cancer Hospital of Shantou University Medical College.

A total of 3,978 patients with oesophageal carcinoma underwent surgical resection between January 2001 and September 2016, including 28 patients with CEC and 3,950 patients with thoracic or abdominal oesophageal cancer. Three hundred and sixty CEC patients without distant metastasis received definitive CRT in the same period. In this study, CEC was defined as a tumour with its centre located between the oesophageal orifice and the sternal notch. Patients with CEC who had concurrent tumours in other segments of the oesophagus were also included in our study.

Medical history was obtained from all patients, and then, they underwent a physical examination. A chest radiograph, Doppler ultrasound examination of the neck; barium meal; contrast-enhanced computed tomography scan of the neck, chest and upper abdomen; oesophagoscopic biopsy; complete blood count; blood biochemistry analyses; and liver and renal function evaluations were also performed. Tumours were staged according to the sixth edition of the American Joint Committee on Cancer (AJCC) staging system for oesophageal cancer.

### Surgical procedure

All patients underwent a total PLE. A transhiatal oesophagectomy without thoracotomy was performed before 2010, while thoracoscopic surgery was used for the thoracic procedures after 2011. Pharyngo-laryngectomy and cervical lymphadenectomy were performed by head and neck surgeons, while thoracoscopic surgery and laparotomy or laparoscopic surgery were conducted by the thoracic surgeons. The reconstruction was simultaneously performed by thoracic surgeons. A gastric tube, which was principally elevated through the posterior mediastinal route, was used for oesophageal reconstruction. In all patients, standard abdominal lymphadenectomy and selective cervical lymphadenectomy were performed. Mediastinal lymphadenectomy was performed for one patient who underwent a thoracoscopic surgery.

### Preoperative therapy

Of the 28 patients with CEC who underwent surgical resection, only 2 patients received scheduled neoadjuvant radiotherapy of a total dose of 40 Gy and underwent oesophagectomy 4 weeks after the radiotherapy. Radiotherapy was delivered using an MV linear accelerator in 2 Gy fractions 5 days a week. Another 8 patients received definitive CRT and underwent salvage surgery after the recurrence of the tumour. A total dose of 60–68 Gy (median 60 Gy) was delivered. Chemotherapy with cisplatin plus 5-fluorouracil was repeated every 3 weeks for 2 to 4 cycles. The time from the termination of CRT to failure was 5 months to 7 years (median, 7 months).

### Postoperative therapy

None of the 10 patients who received preoperative therapy underwent postoperative chemotherapy or radiotherapy. For the other 18 patients who chose surgery as their primary treatment, six of them received adjuvant radiotherapy with a total dose of 50 Gy.

### Follow-up

Patients were followed with a clinical examination every 3 months for the first year, every 6 months for the second year and every 6 to 12 months thereafter. During each follow-up visit, the patients received a clinical evaluation, blood biochemistry examination, ultrasonography exam, and X-ray examination. Computed tomography was performed every 6 months. Endoscopic examinations were performed when necessary. Follow-up was continued up to November 2016 or until death, whichever occurred earlier.

### Statistical analysis

Statistical analyses were performed using the SPSS 18.0 software (SPSS Inc., Chicago, IL, USA). Clinicopathological data between different groups were statistically compared by means of the χ2 test or Fisher’s exact test. Univariate analysis of survival was performed using the Kaplan-Meier method to estimate the survival probabilities, and the log-rank test was used for statistical comparisons. Multivariate analysis was performed to investigate prognostic factors for CEC patients using the Cox proportional hazard regression model with the entry factors of gender, age (≤60 years versus > 60 years), tumour length (≤4 cm versus > 4 cm), tumour cell differentiation, pT category, pN category, pTNM stage, surgical procedure and salvage surgery. All statistical tests performed were two-sided, and a *P*-value less than 0.05 was considered to be statistically significant.
